# Development and Validation of 3D Finite Element Models for Prediction of Orthodontic Tooth Movement

**DOI:** 10.1155/2018/4927503

**Published:** 2018-08-30

**Authors:** Udomsak Likitmongkolsakul, Pruittikorn Smithmaitrie, Bancha Samruajbenjakun, Juthatip Aksornmuang

**Affiliations:** ^1^Orthodontic Section, Department of Preventive Dentistry, Faculty of Dentistry, Prince of Songkla University, Hat Yai, Songkhla, Thailand; ^2^Department of Mechanical Engineering, Faculty of Engineering, Prince of Songkla University, Hat Yai, Songkhla, Thailand; ^3^Prosthodontic Section, Department of Conservative Dentistry, Faculty of Dentistry, Prince of Songkla University, Hat Yai, Songkhla, Thailand

## Abstract

**Objectives:**

The aim of this study was to develop and validate three-dimensional (3D) finite element modeling for prediction of orthodontic tooth movement.

**Materials and Methods:**

Two orthodontic patients were enrolled in this study. Computed tomography (CT) was captured 2 times. The first time was at *T*_0_ immediately before canine retraction. The second time was at *T*_4_ precisely at 4 months after canine retraction. Alginate impressions were taken at 1 month intervals (*T*_0_–*T*_4_) and scanned using a digital scanner. CT data and scanned models were used to construct 3D models. The two measured parameters were clinical tooth movement and calculated stress at three points on the canine root. The calculated stress was determined by the finite element method (FEM). The clinical tooth movement was measured from the differences in the measurement points on the superimposed model. Data from the first patient were used to analyze the tooth movement pattern and develop a mathematical formula for the second patient. Calculated orthodontic tooth movement of the second patient was compared to the clinical outcome.

**Results:**

Differences between the calculated tooth movement and clinical tooth movement ranged from 0.003 to 0.085 mm or 0.36 to 8.96%. The calculated tooth movement and clinical tooth movement at all reference points of all time periods appeared at a similar level. Differences between the calculated and clinical tooth movements were less than 0.1 mm.

**Conclusion:**

Three-dimensional FEM simulation of orthodontic tooth movement was achieved by combining data from the CT and digital model. The outcome of the tooth movement obtained from FEM was found to be similar to the actual clinical tooth movement.

## 1. Introduction

Orthodontic tooth movement has been routinely practiced in clinics, but the orthodontic treatment force is largely unknown. Knowledge of the biomechanical changes in the loaded tissues and the mechanisms of tissue response on an applied force are difficult to study because the stress/strain in a periodontal ligament cannot be measured directly and must be derived from mathematical models.

An initial study attempted to relate tooth movement to an applied force by developing a simple theory that assumed that the force is a constant value with imprecise experimental techniques on human subjects [1]. Other studies in tooth movement and properties of the periodontium were animal experiments [2–8]. This approach has limitations to explain the biomechanics of tooth movement in humans because animal tissues often produce morphological and biomechanical changes unlike human tissues. The development of tissue culture systems to determine the effects of stress on osteoblast cells was reported [9]. Finding the effects of stress at the cellular level has driven much research in an attempt to understand the mechanisms of cell reaction in the process of tooth movement. Several published papers on this issue can be found during the last decade [9–11]. The linking of an application of a continuous load on a tooth crown in an orthodontic force all the way down to the cellular response may provide a much clearer picture to the clinician and improve the biomechanical understanding of the resultant local stress/strain. The methods to study the biomechanics of tooth movement include theoretical mathematical techniques [12], photoelastic systems [13], and laser holographic interferometry [14]. Some methods were effective in predicting the tissue response from the applied load. However, some were ineffective because they examined only the surface stress and were not well validated. Clinically, the observation time of the tooth movement was too long to describe the biomechanics of stress and strain in orthodontics. Besides, the biological cell response in humans has individual variations. Recently, the finite element method (FEM), which was originally used in structural analysis, has been applied in dental biomechanical predictions [15, 16]. The FEM has been used to solve stress-strain problems in the mechanics of solids and structures. This analysis technique has been adopted to study biomaterials and human structures as well. Thus, the biomechanics of orthodontic tooth movement can be analyzed by this method. Although clinical validation of the FEM in orthodontic tooth movement is necessary, it has not been previously reported. The aim of this study was to develop and validate the prediction of three-dimensional (3D) finite element modeling of orthodontic tooth movement.

## 2. Materials and Methods

Two patients were enrolled in this study. They were 18 and 20 years old and attended the Orthodontic Clinic, Faculty of Dentistry, Prince of Songkla University. Orthodontics patients from our clinic, who meet the inclusion criteria and had not previously received any orthodontic therapy, were enrolled in this study. The inclusion criteria were good general health, no medical problems, no signs or symptoms of temporomandibular dysfunction, good oral hygiene, and probing depth values of the entire dentition less than 3 mm. Patients had to be diagnosed as skeletal Class I bimaxillary dentoalveolar protrusion and normodivergent pattern with a plan to extract the maxillary and mandibular first premolars and distalize the canines. The absolute maximum anchorage situation was determined. The research protocol was approved by the Research Ethics Committee of the Faculty of Dentistry, Prince of Songkla University (Project No. EC5804-10-P-HR). For each patient, Roth's prescription preadjusted edgewise brackets (Ormco Corporation, Glendora, CA, USA) with 0.018 × 0.025-inch slots were attached on the incisors, 0.022 × 0.028-inch slots were attached on the posterior teeth, and self-ligating brackets (Ormco Corporation, Glendora, CA, USA) with 0.022 × 0.028-inch slots were attached on the canines after extraction of the first premolars. For anchorage preparation, temporary skeletal anchorage devices (Dentos, Daegu, Korea) were placed on the attached gingiva between the second premolar and first molar. The second premolar through the second molar were tied together with 0.010-inch stainless steel wire and bound with 0.016 × 0.022-inch stainless steel wire to passively engage the tubes and slots of the edgewise appliances. The wires were left in situ for 1 month to become passive before starting to retract the canines. The mechanics for canine distalization consisted of a NiTi-based closed-coil spring (Dentos, Light, Daegu, Korea) tied between the canine and temporary skeletal anchorage device. The NiTi coil springs were activated to obtain 100 grams of force. Each canine was retracted for 4 months. The patients were scheduled for visits every month. At each appointment during the experimental period, the NiTi coil spring was checked and adjusted to ensure that the level of the force was at 100 grams.

**Figure 1:**
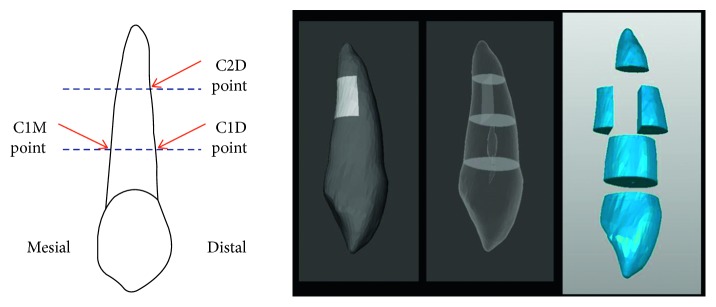
Measurement landmarks in the canine model.

For construction of the 3-dimensional model, the patient's head image was captured 2 times with low-dose dental computed tomography (CT) using a Veraviewepocs (Morita, Tokyo, Japan). The first CT image at *T*_0_ was taken immediately before canine retraction and the last CT image at *T*_4_ was taken after canine retraction precisely at 4 months. The dental CT scan at *T*_0_ was saved in DICOM format and later converted to an initial model. The model between the mesial of the lateral incisor and mesial of the second premolar area included the maxilla, alveolar bone, periodontal ligament, lateral incisor, canine, second premolar, bracket, main archwire, and the temporary skeletal anchorage device. It was constructed by 3D image processing and editing software (ITK-SNAP open-source software). The 3D model of the canines was sectioned into buccal and palatal sides. The root was divided at one-third and two-thirds of the root length. Three points were defined as C1D, C2D, and C1M. C1D and C2D points were on the distal surface of the tooth at one-third and two-thirds coronal to the root, respectively. The C1M point was on the mesial surface of the tooth at one-third coronal to the root (Figure 1).

At each appointment (*T*_0_–*T*_4_), alginate impressions were taken to determine the amount of tooth movement. The models were then scanned using a calibrated digital scanner (R700 Orthodontic 3D Scanner, 3 Shape, Copenhagen, Denmark).

**Figure 2:**
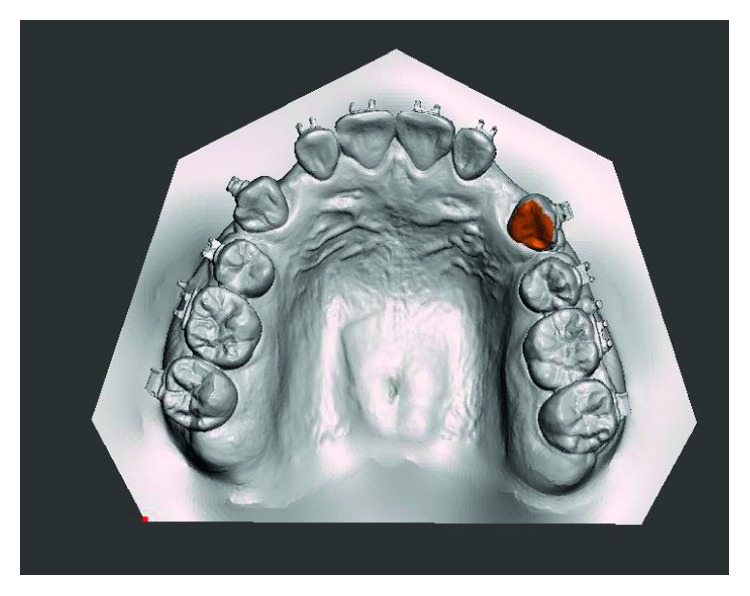
Superimposition of the constructed tooth model and the scanned model using the palatal side of the teeth.

The *T*_1_ constructed model was generated from individually constructed tooth models and periodontal ligament models from the *T*_0_ superimposed over the *T*_1_ scanned dental model based on the best-fit method at the area of palatal side using 3D image processing software (Geomagic Wrap software; www.geomagic.com) (Figure 2). Maxilla from *T*_0_ was simultaneously adjusted to the new position of canine and periodontal ligament. The *T*_2_ and *T*_3_ constructed models were generated with the same method. Finally, the *T*_4_ constructed model was generated from the *T*_4_ dental CT following the *T*_0_ model technique. The *T*_0_, *T*_1_, *T*_2_, and *T*_3_ constructed models were saved in STL format.

**Figure 3:**
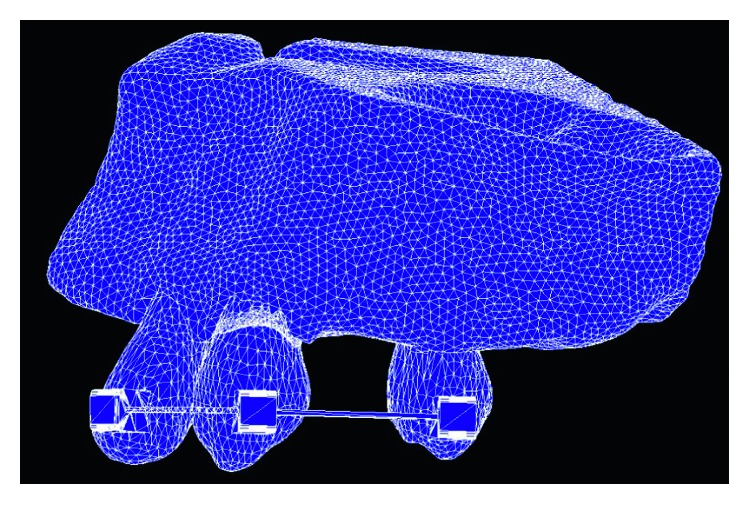
The mesh model of the maxilla, alveolar bone, periodontal ligament, lateral incisor, canine, second premolar, bracket, and main archwire.

To analyze the 3D solid model, the program required the numerical dimensions of the object, and it must be able to visualize how these dimensions relate to lines and curves. Constructed models were used to generate finite element mesh and exported as a mesh model. The *T*_0_, *T*_1_, *T*_2_, and *T*_3_ constructed models were meshed by finite element analysis preprocess and postprocess software (MSC Patran; MSC Software, Inc., USA). The tetrahedral element was used in mesh generation. The resultant volumetric model consisted of approximately 100,000 elements and 170,000 nodes (Figure 3). The mesh model was imported into the 3D finite element software (MSC Marc; MSC Software, Inc., USA). Mechanical properties of each tissue and materials are shown in Table 1. The model imitated the clinical situation by applying force at the canine bracket. The direction was set to the temporary skeletal anchorage device in the mesh model, and the magnitude was 100 grams of force. The boundary condition between bracket and wire was touch contacted that the bracket can slide along the archwire. The program calculated stress from the effect of applied force, boundary conditions, and mechanical properties of each tissue.

**Figure 4:**
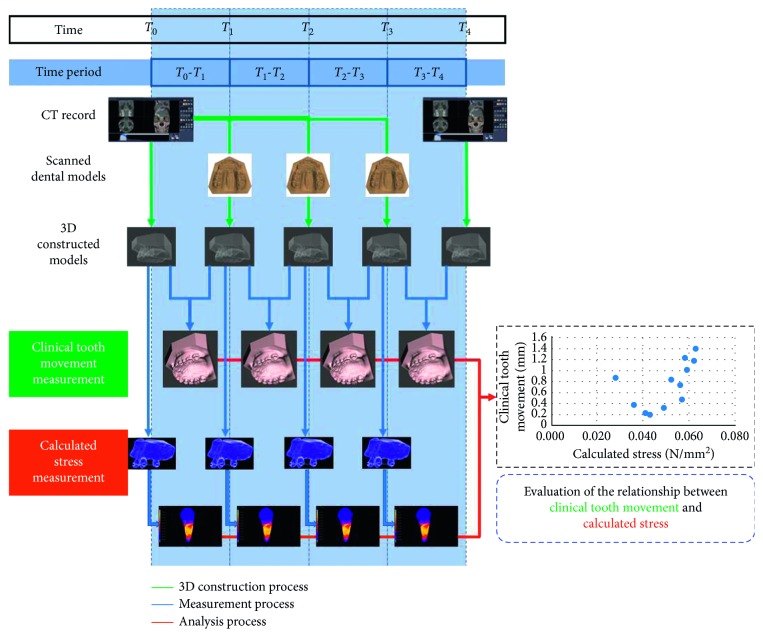
Overview of the experimental steps and measurements.

The two measured parameters were clinical tooth movement and calculated stress at the C1D, C2D, and C1M points. Clinical tooth movement was determined from the difference of the measurement points on the superimposed model. The distance measurements were repeated at 2-week intervals. The method error was calculated using Dahlberg's formula [17]. The stress was determined by the FEM. The 3D models and analyses in each period are shown in Figure 4. To create a formula for the prediction of tooth movement, data from the first patient were used to analyze the tooth movement pattern. Data of tooth movement in each visit were plotted showing the relationship between tooth movement and stress. All data and stress from the finite element model of the first patient were used to create the pattern prediction formula which was subsequently used in the second patient.

The dental CT data and the scanned dental model of the other patient were processed with the same method as the first patient. The formula from the first patient was used to calculate the orthodontic tooth movement which was then compared with the clinical outcome.

## 3. Results and Discussion

**Figure 5:**
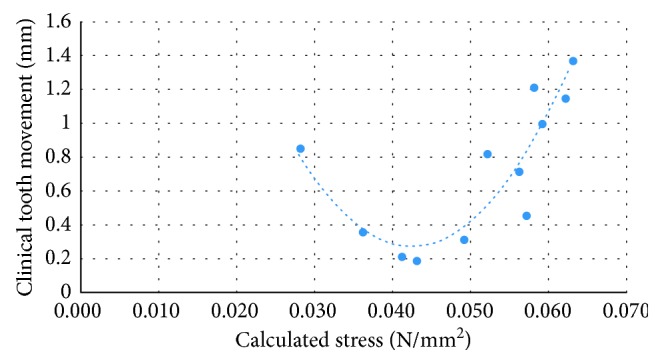
Relationship between the calculated stress and clinical tooth movement of the first orthodontic patient.

The results of the calculated stress and clinical tooth movement obtained from the first patient are shown in Table 2. The ranges of the calculated stress and clinical tooth movement were 0.028 to 0.063 MPa and 0.194 to 1.378 mm, respectively. The data from the calculated stress and clinical tooth movement were plotted (Figure 5). The relationship between the calculated stress and clinical tooth movement was matched to a quadratic trend. The fitting formula can be written as Equation (1), where *Y* is the tooth movement (mm) and *X* is the stress (N/mm^2^):

(1) $$\begin{array}{c} Y=2960X^{2}-254.56X+5.667. \end{array}$$

**Figure 6:**
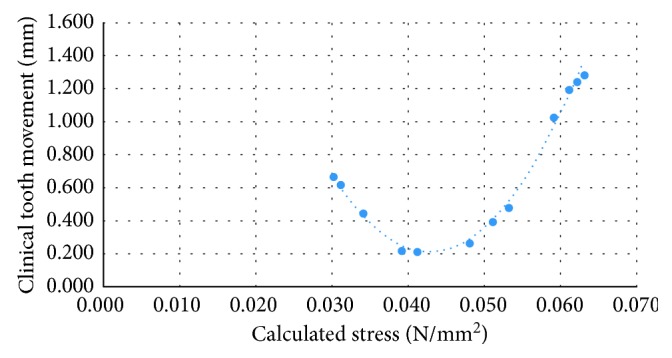
Relationship between the calculated stress and clinical tooth movement of the second orthodontic patient.

The results of the calculated stress, calculated tooth movement, and clinical tooth movement of the second patient are shown in Table 3. The results revealed that the range of calculated stress, calculated tooth movement, and clinical tooth movement were 0.030 to 0.063 MPa, 0.206 to 1.378 mm, and 0.219 to 1.296 mm, respectively. The differences between the calculated tooth movement and clinical tooth movement ranged from 0.003 to 0.085 mm or 0.36 to 8.96%. The calculated tooth movement and clinical tooth movement at all reference points of all time periods appeared to be at similar levels. Differences in the values were less than 0.1 mm. The data from the calculated stress and clinical tooth movement were plotted (Figure 6).

Orthodontic tooth movement of the first patient was studied and simulated to develop a mathematical formula to represent the relationship between stress and the amount of tooth movement. Orthodontic tooth movement of the second patient was predicted using the formula from the first patient and then compared with the clinical outcome.

Two patients with absolute maximum anchorage were included in this study. Therefore, a skeletal anchorage device was used to reduce the complexity of mimicking the orthodontic tooth model. The first point of loading was fixed at the skeletal anchorage device. The loading force and direction were therefore virtually stable between each month. In addition, the skeletal anchorage device was employed as a landmark in the superimposition process and for the measurements. The NiTi-based closed-coil spring was attached to distalize the canine. Since the properties of NiTi generated a continuous force with a low decay rate [18], the magnitude of force could be controlled at each visit. Our previous study proved that 3D constructed models could be accurately created using the scanned plaster dental model combined with the original CT scan data [19]. To reduce the CT dose, *T*_1_, *T*_2_, and *T*_3_ were therefore evaluated using only the impression method.

The canine model was segmented into six pieces to reduce systematic error in the measurements. The measurement landmarks were at the point angles of the segments. Our previous experiment was performed to validate landmarks of the 3D model [19]. The results showed that the segmental tooth model was effective for evaluating the tooth position. In this study, Dahlberg's error of distance measurement was less than 0.001 mm. The results of this study confirmed that the segmented model could improve reproducibility and generate less error.

The results in Table 2 show that the stress ranged from 0.028 to 0.063 MPa. In a study by Rudolph et al., a force of 0.25 N was used to tip the upper central incisor. The results found that the maximum stress was 0.00245 MPa at the cervical area and approximately 0.00765 to 0.00122 MPa in the one-third to two-thirds area [20]. In another previous study that used a force of 0.5 N, stress in the crestal bone was found to range from 0.0187 to 0.0560 MPa [21]. The results of stress in this study were higher than those of the previous studies due to the higher force used. A force magnitude of 1 N was applied in the present experiment which was 2 to 4 times higher than the previous experiments. The finite element model in this study was composed of more than 170,000 nodes and 100,000 elements (10-node quadratic tetrahedron). The model consisted of cortical bone, cancellous bone, tooth, and periodontal ligament which could represent the shape and type of each tissue similar to the real situation.

A previous study reported that stress was found to be a factor that affected tooth movement [22]. Kojima et al. reported the equations between stress and bone resorption to demonstrate tooth movement through the alveolar bone [23]. This present study revealed the relationship between stress and tooth movement directly and the relationship was validated by the clinical outcome. The relationship between stress and tooth movement is represented by the plot shown in Figure 5. It was found that the relationship between stress and tooth movement was nonlinear as demonstrated in previous studies [24–26]. Tooth movement initially occurred at a low level of stress. The rate of tooth movement decreased until it passed the stress threshold and then the tooth movement increased rapidly (Figure 5). This can be described by the pressure-tension theory; that is, the optimal force could activate the biological mechanism and then the tooth would move [27, 28]. As the stress increased, blood flow would consequently decrease. Blood flow serves as the source of mediation in biological mechanisms. The amount of tooth movement therefore decreased in the middle of the movement pattern. If the stress continuously increased, blood flow would be cut-off and the tissue adjacent to this area would become necrotic. A biological mechanism would also occur in the alveolar bone and the amount of tooth movement would increase based on the extent of the necrotic zone. However, the side effects of a high magnitude of force, such as root resorption, could not be simulated in this study.

**Figure 7:**
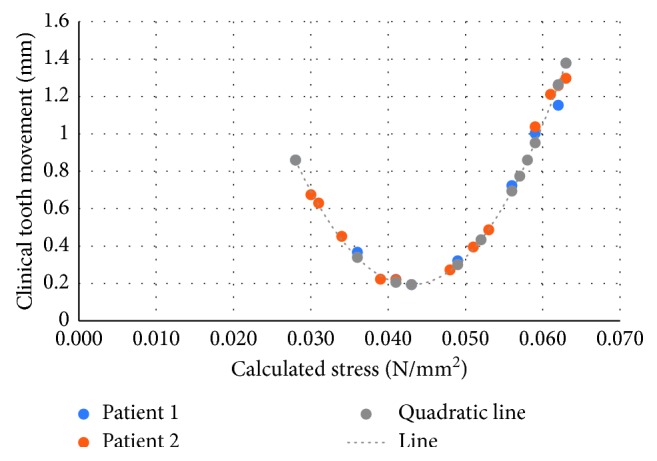
Relationship between the calculated stress and clinical tooth movement of the first and second orthodontic patient and quadratic line of the equation.

The differences between the predicted tooth movement and clinical tooth movement of the second orthodontic patient ranged from 0.03 to 0.086 mm or 0.36 to 8.95%. The data obtained from the calculated tooth movement were found to be allied with the clinical tooth movement. However, the prediction of tooth movement by this method is suitable for a one-month period. A series of prediction models should be performed to predict tooth movement for time periods longer than one month because the stress would change during treatment and would affect the amount of tooth movement. The other factor that affects the outcome is individual tissue response [29]. Although data from the second patient seem to be in better agreement with the equation, the error in the data of the first patient occurred from one point of measurement, C1M, which might affect the multiple processes. When the data of C1M for the first patient was excluded, the agreement in both patients was similar (Figure 7). However, the present study revealed similar results between the two patients. The equation created from the first patient could be used to predict the clinical outcome of the second patient (Table 3).

The FEM presented in this study would be an alternative method to simulate orthodontic tooth movement. This could assist treatment planning by predicting the outcome of clinical treatment and assist orthodontists in choosing a treatment option for the best outcome. Researchers should be able to use this finite element prediction to test various mechanics, and materials in different situations for orthodontic plans before a clinical trial which traditionally takes a long time and usually involves ethical issues. However, this technique requires several procedures and an investment of time for the 3D modeling process. The evolution of computer technology may facilitate simpler 3D modeling methods in the near future. The FEM processing protocol should be developed further in terms of saving time and user friendliness for general orthodontists.

The major limitation of this research is the small sample size due to the difficulty in 3D model production and analysis, which take long periods of time [30]. Orthodontic tooth movement is a biological response depending upon the individual. Affirmation of FEM results to clinical results therefore should be performed in a greater quantity of sample size. However, this precursory research has delivered positives results, which can be continuously researched.

## 4. Conclusions

Within the limitation of this study, it can be concluded that the 3D FEM simulation of orthodontic tooth movement can be achieved by combining data from a dental CT and a digital model. This technique provided acceptable accuracy. The outcome of tooth movement obtained from the FEM was found to be similar to the actual clinical tooth movement.

## Figures and Tables

**Table 1:** Mechanical properties of the materials.

Tissue	Material	Young's modulus (N/mm^2^)	Poisson's ratio
Maxilla	Cortical bone	13,800	0.26
	Cancellous bone	345	0.31

Lateral incisor	Tooth	20,000	0.15
Canine			
Second premolar			

Periodontal ligament	Periodontal ligament	0.68	0.49

Bracket	Stainless steel	210,000	0.30
Main archwire			

**Table 2:** Calculated stress and clinical tooth movement of the first orthodontic patient.

Point	Time	Calculated stress (N/mm^2^)	Time	Clinical tooth movement (mm)
C1M	*T*_0_	0.062	*T*_0-1_	1.153
C1D		0.059		1.003
C2D		0.041		0.221

C1M	*T*_1_	0.058	*T*_1-2_	1.221
C1D		0.063		1.378
C2D		0.043		0.194

C1M	*T*_2_	0.052	*T*_2-3_	0.827
C1D		0.056		0.723
C2D		0.028		0.859

C1M	*T*_3_	0.057	*T*_3-4_	0.464
C1D		0.049		0.321
C2D		0.036		0.367

**Table 3:** Calculated stress, calculated tooth movement, and clinical tooth movement of the second orthodontic patient.

Point	Time	Calculated stress (N/mm^2^)	Time	Calculated tooth movement (*F*) (mm)	Clinical tooth movement (*C*) (mm)	Difference (|*F *−* C*|) (mm (%))
C1M	*T*_0_	0.063	*T*_0-1_	1.378	1.296	0.082 (5.95)
C1D		0.061		1.153	1.211	0.058 (5.03)
C2D		0.039		0.241	0.223	0.018 (7.61)

C1M	*T*_1_	0.062	*T*_1-2_	1.263	1.258	0.005 (0.36)
C1D		0.059		0.952	1.037	0.085 (8.96)
C2D		0.041		0.206	0.219	0.013 (6.39)

C1M	*T*_2_	0.053	*T*_2-3_	0.490	0.487	0.003 (0.61)
C1D		0.051		0.383	0.395	0.012 (3.01)
C2D		0.048		0.268	0.272	0.004 (1.49)

C1M	*T*_3_	0.030	*T*_3-4_	0.694	0.674	0.020 (2.92)
C1D		0.031		0.620	0.630	0.010 (1.57)
C2D		0.034		0.434	0.451	0.017 (3.97)

## Data Availability

The data used to support the findings of this study are available from the corresponding author upon request.
